# Anterior cruciate ligament remnant tissue harvested within 3-months after injury predicts higher healing potential

**DOI:** 10.1186/s12891-015-0855-0

**Published:** 2015-12-18

**Authors:** Shurong Zhang, Tomoyuki Matsumoto, Atsuo Uefuji, Takehiko Matsushita, Koji Takayama, Daisuke Araki, Naoki Nakano, Kanto Nagai, Tokio Matsuzaki, Ryosuke Kuroda, Masahiro Kurosaka

**Affiliations:** Department of Orthopaedic Surgery, Kobe University Graduate School of Medicine, 7-5-1 Kusunoki-cho, Chuo-ku Kobe, 650-0017 Japan

**Keywords:** Anterior cruciate ligament, Surgical timing, Multivariate logistic regression

## Abstract

**Background:**

No study has examined the possible factors associated with different characteristics of stem-like cells derived from anterior cruciate ligament (ACL) remnants. And the purpose of the study is to elucidate whether demographic factors are associated with healing potential of stem-like cells derived from the ACL remnants tissue.

**Methods:**

Thirty-six ACL remnants were harvested from patients who received primary arthroscopic ACL reconstruction. Interval from injury to surgery, age, sex, and combined meniscal or chondral injuries were analyzed. Cells were isolated from remnant tissues and their healing potential was evaluated by: 1) characterization of surface markers (CD34, CD44, CD45, CD146, CD29, and Stro-1), 2) cell expansion, 3) osteogenic differentiation, and 4) endothelial differentiation. Finally, using multivariable logistic regression to evaluate the relation between demographic factors and healing potential parameters. Adjusted odds ratios (OR) were calculated, and the significant difference was set at *p* < 0.05.

**Results:**

ACL remnant tissue harvested less than 90 days after injury predicted higher fractions of stem-like cells [CD34+ (OR = 6.043, *p* = 0.025), CD44 + (OR = 8.440, *p* = 0.011), CD45+ (OR = 16.144, *p* = 0.015), and CD146+ (OR = 9.246, *p* = 0.015)] and higher expansion potential (passage 3: OR = 9.755, *p* = 0.034; passage 10: OR = 33.245, *p* = 0.003). Regarding osteogenic differentiation, higher gene expression of Osteocalcin (OR = 22.579, *p* = 0.009), Alkaline phosphatase (OR = 6.527, *p* = 0.022), and Runt-related transcription factor 2 (OR = 5.247, *p* = 0.047) can also be predicted. Younger age predicted higher CD34+ levels (20 ≤ age <30 years, OR = 2.020, *p* = 0.027) and higher expansion potential at passage 10 (10 ≤ age <20 years, OR = 25.141, *p* = 0.026). There was no significant relation found between meniscal or chondral injuries and ACL healing potential.

**Conclusion:**

Our results indicated that the ACL remnant tissue harvested within 3-months after injury yields higher healing potential, suggesting early surgical intervention may achieve better clinical results.

## Background

The anterior cruciate ligament (ACL) is often injured, which predisposes the knee to subsequent injuries or early onset of osteoarthritis (OA). Although ruptured ACLs can be reconstructed, good results are not guaranteed, because of graft failure and recurrent instability, as reported 0.7–10 % of patients [1, 2]. Therefore, in addition to enhancing surgical techniques, intrinsic healing potential of the ACL should not be overlooked [3].

A tibial stump connecting [4] is often observed in acute or sub-acute ACL injury during arthroscopy, indicating that ACL fibers might have intrinsic healing potential. A rich supply of CD 34+ stem/progenitor cells was primarily found in blood vessels [5–8], and its healing potential was reinforced by several publications consecutively [9–12]. Since Matsumoto et al. reported that CD34+ cells derived from human ACL ruptured tissue exhibited higher proliferation and multi-lineage differentiation potential [10], successive studies supported that these cells or the ruptured tissue may contribute to the tendon-bone healing as well as to the reduction of tunnel enlargement [13–15].

However, controversies exist [16] on these results because a superior clinical outcome cannot be assured by the remnant preserving techniques compared to those obtained with standard ACL reconstruction techniques [17, 18]. We believe that some other factors might be involved in the healing potential.

Therefore, the purpose of this study was to identify whether demographic factors such as interval between injury and surgery, age, sex, and combined meniscal or chondral injuries were associated with characteristics of ACL remnant-derived stem-like cells in the healing potential of reconstructed ACL. The hypothesis was that one or more factors would be associated with the characterization, proliferation, and multi-lineage differentiation potential of ACL remnant-derived stem-like cells.

## Methods

The Institutional Review Board of Kobe University approved this study. Written informed consent was obtained from all the subjects recruited in the present study.

### Subjects

Patients who had undergone primary arthroscopic ACL reconstruction and those younger than 50 years were included. ACL remnants from ACL revision surgery or combined injury involving medial/lateral collateral ligament or posterior cruciate ligament were excluded from the study. Thirty-six ACL remnant tissues were harvested by an arthroscopic surgeon with more than 10 years’ experience in arthroscopy. The interval between injury and surgery, age at injury, sex, and combined meniscal or chondral injuries were selected as risk factors. The interval between injury and surgery was categorized as < 90 days and ≥ 90 days. Age was categorized as the 10′ group (10 ≤ age <20 years), the 20′ group (20 ≤ age <30 years), and over 30′ group (≥30 years).

### Cell isolation

The ACL remnant tissues were harvested from the rupture site of 1.0 cm^3^ in size, preserved in the sterile saline solution on ice, minced into 1–2 mm small particles, washed by phosphate-buffered saline (PBS) three times, and then digested with 0.4 % collagenase type II (0.4 % w/v; Worthington Biochemical Corporation, Lakewood, New Jersey) for two hours in a 37 °C 5 % CO_2_ incubator. The suspension was washed with PBS and centrifuged at 1500 rpm for 10 min, and the supernatant was discarded; the cells were suspended in PBS, and then filtered through a 70-μM nylon cell strainer (Becton Dickinson [BD], Franklin Lakes, New Jersey). Later, the cells were washed with PBS twice, cultured in standard Dulbecco’s Modified Eagle’s medium (DMEM) supplemented with 10 % fetal bovine serum (FBS) and 100 U/mL penicillin/streptomycin (Sigma-Aldrich) at a density of 1 × 10^5^ in 75 cm^2^ collagen-coated flask, then incubated in 37 °C 5 % CO_2_ incubator. The medium was changed every 3 days, and passaged the cells when they reached 70–80 % confluent.

### ACL remnant tissue-derived cell characterization

Flow cytometry assays were performed to characterize ACL-derived cells. The in vitro-isolated cells were washed three times with 0.5 % bovine serum albumin-PBS for 5 min to prepare single-cell suspensions with density of 1 × 10^6^ cells/mL and perform the assay. Fluorescein isothiocyanate-labeled primary antibody was diluted in mouse serum at a ratio of 1:10 (Sigma-Aldrich) and was incubated with cells for 30 min on ice. Isotype control was defined as nonspecific mouse IgG substituting for the primary antibody. Facial Action Coding System (FACS) was performed by FACS Calibur (BD Bioscience Pharmingen) and analyzed by CellQuest (BD Pharmingen) software. The expression of cluster of differentiation markers CD34/PE, CD146/FITC, CD45/allophycocyanin (APC), CD44/FITC, CD29/PE, and Stro-1/Alexa Fluor647 (BD Bioscience Pharmingen, San Diego, California) were evaluated in this study.

### Expansion potential assay

The expansion potential was calculated by population doublings (PDs). Cells were cultured in 75 cm^2^ collagen-coated flasks, and passaged every 7 days at the cell density of 1.0 × 10^5^/flask. The number of PDs of each passage was calculated as log_2_ (N/1.0 × 10^5^).

### Multi-lineage differentiation assessment

#### Osteogenic differentiation

**Fig. 1:**
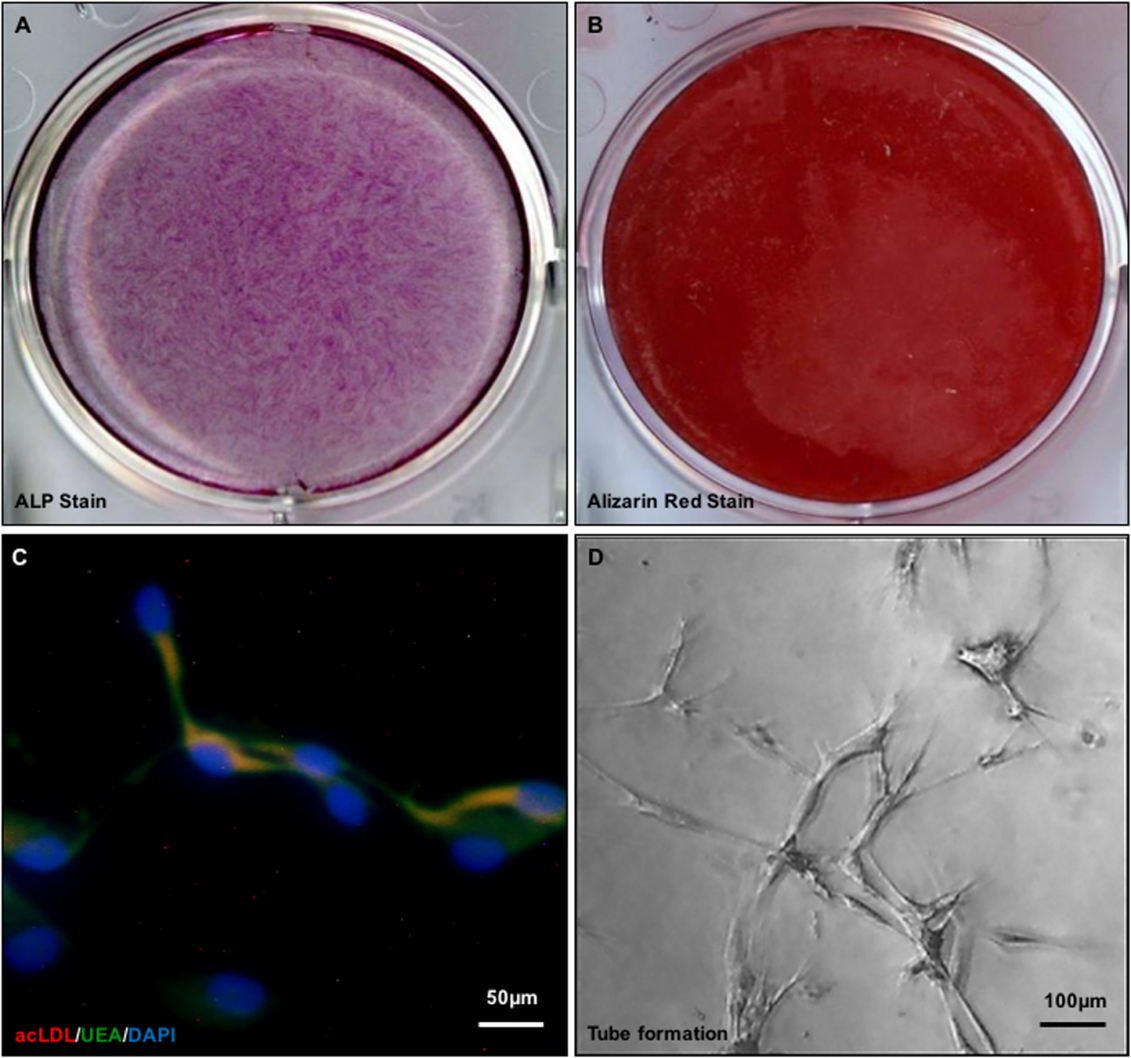
The figure showed ALP staining (a), Alizarin red S staining (b), fluorescence microscopic view of ACL remnant derived cells' Dil-Labeled acLDL uptake and binding to Ulex Europaeus Lectin (c), and endothelial tubular formation (d) of the ACL-derived cells

In line with a previously reported osteogenic differentiation assessment [19, 20], a density of 1.0 × 10^5^/well, four replicated ACL-derived cells were treated with an osteogenic medium, known as the α-minimum essential medium (Invitrogen, Carlsbad, California) supplemented with 10 % FBS, 100 U/mL penicillin/streptomycin solution (Sigma-Aldrich), 0.1 mM dexamethasone (Sigma-Aldrich), 50 mM ascorbate-2-phosphate (Sigma-Aldrich), and 10 mM β-glycerophosphate (Sigma-Aldrich). To assess the osteogenesis, the cells were fixed in 1 mL of ice-cold 70 % ethanol for 1 h at 4 °C and incubated with alkaline phosphatase (ALP) dye (Fig. 1a) for 14 days. The same procedure was performed after 21 days with alizarin red S dye (Fig. 1b). Total RNA was isolated from cells on day 21 to analyze expression of *Osteocalcin* (F:TGTGAGCTCAATCCGGACTGT; R: CCGATAGGCCTCCTGAAAGC), *Alkaline phosphatase (ALP)* (F: ATGGGATGGGTGTCTCCACA; R: CCACGAAGGGGAACTTGTC), and *Runt-related transcription factor 2* (Runx2) (F: CCAGATGGGACTGTGGTTACTG; R: TTCCGGAGCTCAGCAGAATAA) using quantitative reverse transcription-real-time polymerase chain reaction (qRT-PCR).

### Endothelial differentiation

A density of 1.0 × 10^4^/well, 4 replicates cells were cultured in an endothelial growth medium-2 (EGM-2) bullet kit (endothelial cell basic medium, hydrocortisone, fibroblast growth factor-basic, vascular endothelial growth factor, recombinant human long R3 insulin-like growth factor-1 (R3-IGF-1), ascorbic acid, epidermal growth factor (EGF), Gentamicin (GA; Amphotericin-B)-1000, and heparin) supplemented with 10 % FBS, and incubated at 37 °C in 5 % CO_2_ for 1 week.

To determine the cellular ability to uptake 1,1′-dioctadecyl-3,3,3′,3′ -tetramethylindocarbocyanine (DiI)-labeled acetylated low-density lipoprotein (acLDL) (Biomedical Technologies, Staughton, Massachusetts) and binding potential to Ulex europaeus lectin (Molecular Probes, Eugene, Oregon), the cells were first incubated with DiIacLDL (10 mg/mL) at 37 °C for 4 h, and then fixed with 1 % paraformaldehyde for 10 min. Subsequently, cells were continuously incubated with FITC–Ulex europaeus lectin (10 mg/mL) for 1 h. The slides were mounted by 4′,6-diamidino-2-phenylindole (DAPI) mounting medium and viewed using an inverted fluorescence microscope (Fig. 1c).

Further, endothelial tubular formation was studied in Matrigel cultures. Briefly, 1.0 × 10^4^ cells cultured in endothelial basal medium-2 (EBM-2) were seeded onto 48-well plates coated with Matrigel (BD Biosciences, San Jose, California). After 48 h cultured at 37 °C, the total tube length was calculated from 3 randomly selected low-power fields under a microscope (Fig. 1d).

Total RNA was harvested on day 7 from the cells in monolayer culture–maintained endothelial cell culture EGM-2. Expressions of endothelial *VE-cadherin* (VE-cad, F: GGGAGACCACGCCTCTGTC; R: ACTGAACCTGACCGTAGAGGAGGCCCTGGGCATCTC) and *cluster of differentiation 31(CD31*) (F: GGTTCTGAGGGTGAAGGTGA; R: TTGCAGCACAATGTCCTCTC) were analyzed using qRT-PCR.

### RNA extraction and quantitative reverse transcription-real-time polymerase chain reaction

For qRT-PCR, total RNA was extracted from cells using TRIzol reagent (Invitrogen) and an RNeasy mini kit (Qiagen, Hilden, Germany) with on-column DNase digestion. Later, RNA was reversed-transcribed to complementary deoxyribonucleic acid (cDNA) using the RevertAid First Strand cDNA Synthesis Kit (Applied Biosystems, Foster City, California). The cDNA of each sample was amplified in a 20-μL PCR reaction mixture containing SYBR Green Master Mix reagent (Applied Biosystems, Tokyo, Japan) on the ABI Prism 7500 Sequence Detection System (Applied Biosystems). Housekeeping *Glyceraldehyde 3-phosphate dehydrogenase (GAPDH)* (F: ATGGGGAAGGTGAAGGTCG; R: GGGGTCATTGATGGCAACAATA) was used as calibrator, and sequences of other primers for gene were obtained, as described previously. The following cycling conditions were applied: denaturation at 95 °C for 10 min, 40 cycles at 95 °C for 15 s, optimal annealing temperature for 20 s, 72 °C for 30 s, and 60 °C to 95 °C with a heating rate of 0.5 °C/s. The relative expression level of gene of interest was normalized to GAPDH and was calculated according to the 2-ΔΔCT formulas. For control, the cells were cultured with growth medium.

### Statistical analysis

Descriptive statistical analysis was performed for demographic factors. The data were expressed as mean ± standard error.

Predictor variables analyzed in the study were tested as follows: the interval between injury and surgery (≥90 days category was used as a reference), sex (male sex was used as reference), age at injury (>30′ group was used as reference), meniscal injury, chondral injury, and combined meniscal or chondral injuries (no meniscal or chondral injuries group was used as reference). Since the number of chondral injury was too small to reach the efficacy, the the combined meniscal or chondral injuries was evaluated.

The characteristics of ACL-derived cells (CD34+, CD44+, CD45+, CD146+, CD29+, and Stro-1+), expansion potential (passage 3 and passage 10), osteogenic differentiation (ALP activity, mRNA gene expression of osteocalcin, ALP, and Runx2), and endothelial differentiation (tube length, mRNA gene expression of VE-cad, and CD31) were tested using multivariate logistic regression for binary outcomes. Since all the original data were presented linearly, the median of each parameter was calculated, and data were categorized into a high group (above the median) and low group (below the median) [21]. Adjusted odds ratios (ORs) and 95 % confidence intervals (CIs) were calculated for each predictor variable. Results were considered statistically significant when the null value (1.00) was absent from the CI or *P* < 0.05. SPSS 21.0 software (SPSS, Inc., Chicago, IL, USA) was applied for the statistical analysis.

## Results

### Demographic characteristics

Sample characteristics are presented in Table 1. A total of 36 patients were recruited into this study. Eighteen patients underwent surgery less than 90 days after the injury (mean, 48.9 ± 21.1 days), whereas 18 patients underwent surgery later than 90 days (mean, 182.4 ± 91.0 days). The age range of the subjects was from 14 to 46 (mean, 24.50 ± 7.97 years). In all, 12, 13, and 11 subjects were included into the 10′, 20′, and >30’s group, respectively. Seventeen men and nineteen women were included. There were 18 cases of meniscal injury and 3 cases of chondral injury reported during the surgery, one patient was reported to have both meniscal and cartilage injury. The relation was evaluated as meniscal injury, chondral injury, and combined meniscal and chondral injury.

**Table 1:** Demographic characteristics

Sex (male:female)	17:19
Age, mean ± SD, year	
10 ≤ age < 20, *n* = 12	16.58 ± 1.68
20 ≤ age < 30, *n* = 13	23.71 ± 2.78
≥ 30, *n* = 11	35.10 ± 4.79
Interval from injury to surgery, day	
< 90 days, *n* = 18	48.9 ± 21.1
≥ 90 days, *n* = 18	182.4 ± 91.0
Meniscal injury, case	18
Chondrl injury, case	3
Meniscus injury or cartilage degeneration, case	20

### Predictors of risk factor

Logistic regression results for characterization of ACL-derived cells, expansion potential, and differentiation potential of osteogenesis and endotheliogenesis are presented in Tables 2, 3, 4, and 5.

**Table 2:** Multivariate association between factors in the CD34+, CD 44+, CD 45+, CD 146+, CD29+, and Stro-1+ (*p < 0.05)

	CD34+			CD44+			CD45+			CD146+			CD29+			Stro-1+		
	*p*	OR	95 % CI	*p*	OR	95 % CI	*p*	OR	95 % CI	*p*	OR	95 % CI	*p*	OR	95 % CI	*p*	OR	95 % CI
Interval from injury to surgery^b^, < 90 days	0.025*	6.043	1.259–28.995	0.011*	8.440	1.615–44.111	0.015*	16.144	1.701–153.212	0.015*	9.246	1.541–55.461	0.369	1.982	0.446–8.819	0.059	4.559	0.944–22.020
10 ≤ age <20 year-old^c^	0.957	1.056	0.144–7.755	0.996	0.994	0.124–7.985	0.773	1.370	0.161–11.643	0.416	0.438	0.060–3.198	0.960	1.048	0.167–6.572	0.112	6.244	0.679–40.503
20 ≤ age <30 year-old^c^	0.027*	2.020	1.413–26.512	0.795	1.370	0.127–14.775	0.593	1.954	0.189–20.215	0.178	0.176	0.016–1.888	0.593	0.569	0.071–4.575	0.577	1.919	0.194–18.972
Sex^a^ Female	0.683	0.692	0.118–4.057	0.593	0.604	0.097–3.780	0.092	7.535	0.717–79.179	0.139	4.148	0.629–27.374	0.218	0.368	0.075–1.809	0.843	1.194	0.207–6.897
Meniscal injury^d^	0.626	0.716	0.187–2.744	0.409	0.563	0.143–2.206	0.069	0.269	0.066–1.106	0.409	0.563	0.143–2.206	0.616	0.711	0.188–2.691	0.406	0.566	0.148–2.168
Chondral injury^d^	0.433	2.714	0.223–32.992	0.327	3.500	0.286–42.769	0.761	0.679	0.056–8.248	0.327	3.500	0.286–42.769	0.554	2.125	0.175–25.775	0.688	0.600	0.049–7.283
Combined meniscal or chondral injuries^d^	0.549	0.667	0.177–2.513	0.593	0.692	0.180–2.668	0.117	0.333	0.084–1.318	0.593	0.692	0.180–2.668	0.503	0.636	0.169–2.391	0.549	0.667	0.177–2.513

^a^Use “male” category as reference

^b^Use “interval more than 90 days” category as reference

^c^Use “age more than 30-year old” category as reference

^d^Use “no meniscal or chondral injuries” category as reference

**Table 3:** Multivariate association in expansion potential (*p < 0.05)

	Passage 3			Passage 10		
	*p*	OR	95 % CI	*p*	OR	95 % CI
Interval from injury to surgery^b^, < 90 days	0.034*	9.755	1.187–80.186	0.003*	33.245	3.245–342.631
10 ≤ age <20 year-old^c^	0.210	4.939	0.407–59.879	0.026*	25.141	1.484–426.057
20 ≤ age <30 year-old^c^	0.521	2.250	0.189–26.831	0.339	3.528	0.267–46.676
Sex^a^ Female	0.576	1.794	0.232–13.909	0.230	3.156	0.483–20.619
Menical injury^d^	0.632	1.400	0.354–5.542	0.406	1.768	0.461–6.775
Chondral injury^d^	0.837	1.300	0.107–15.836	0.492	0.417	0.034–5.057
Combined meniscal or chondral injuries^d^	0.593	1.444	0.375–5.566	0.334	1.929	0.509–7.311

^a^Use “male” category as reference

^b^Use “interval more than 90 days” category as reference

^c^Use “age more than 30-year old” category as reference

^d^Use “no meniscal or chondral injuries” category as reference

**Table 4:** Multivariate association in osteogenic differentiation (*p < 0.05)

	ALP activity			Osteocalcin mRNA			ALP mRNA			Runx2 mRNA		
	*p*	OR	95 % CI	*p*	OR	95 % CI	*p*	OR	95 % CI	*p*	OR	95 % CI
Interval from injury to surgery^b^, < 90 days	0.263	2.416	0.515–11.337	0.009*	20.579	2.119–199.882	0.022*	6.527	1.308–32.557	0.047*	5.247	1.023–26.922
10 ≤ age <20 year-old^c^	0.895	0.884	0.142–5.491	0.384	2.683	0.290–24.782	0.962	0.954	0.141–6.481	0.592	0.600	0.092–3.895
20 ≤ age <30 year-old^c^	0.240	0.277	0.033–2.356	0.909	1.155	0.097–13.787	0.742	1.420	0.176–11.451	0.480	0.463	0.055–3.924
Sex^a^ Female	0.030*	12.831	1.279–128.746	0.850	0.831	0.123–5.606	0.885	1.131	0.212–6.041	0.550	0.601	0.113–3.191
Meniscal injury^d^	0.218	2.400	0.596–9.670	0.198	2.600	0.606–11.152	0.360	1.920	0.475–7.766	0.803	1.200	0.287–5.021
Chondral injury^d^	0.000	0.000	0.000–0.000	1.000	1.000	0.081–12.270	0.000	0.000	0.000–0.000	0.913	1.150	0.093–14.188
Combined meniscal or chondral injuries^d^	0.402	1.800	0.455–7.127	0.346	2.000	0.473–8.462	0.588	1.467	0.367–5.858	0.519	1.615	0.376–6.940

^a^Use “male” category as reference

^b^Use “interval more than 90 days” category as reference

^c^Use “age more than 30-year old” category as reference

^d^Use “no meniscal or chondral injuries” category as reference

**Table 5:** Multivariate association in endothelial differentiation (*p < 0.05)

	Tube length			VE-cadherin mRNA			CD31 mRNA		
	*p*	OR	95 % CI	*p*	OR	95 % CI	*p*	OR	95 % CI
Interval from injury to surgery^b^, < 90 days	0.495	2.409	0.193–30.046	0.836	0.824	0.132–5.156	0.073	7.337	0.832–64.742
10 ≤ age <20 year-old^c^	0.430	2.659	0.234–30.160	0.317	3.118	0.337–28.884	0.504	0.438	0.039–4.924
20 ≤ age <30 year-old^c^	0.966	0.690	0.064–7.429	0.781	1.421	0.120–16.863	0.620	0.494	0.031–8.001
Sex^a^ Female	0.190	4.987	0.452–55.045	0.265	0.335	0.049–2.293	0.458	2.245	0.265–19.001
Meniscal injury^d^	0.848	0.857	0.178–4.126	0.198	2.600	0.606–11.152	0.862	1.125	0.298–4.241
Chondral injury^d^	0.547	0.458	0.036–6.789	0.000	0.000	0.000–0.000	0.619	0.531	0.044–6.444
Combined meniscal or condral injuries^d^	0.966	1.010	0.214–4.674	0.346	2.000	0.473–8.462	0.765	0.818	0.219–3.056

^a^Use “male” category as reference

^b^Use “interval more than 90 days” category as reference

^c^Use “age more than 30-year old” category as reference

^d^Use “no meniscal or chondral injuries” category as reference

### Interval between injury and surgery

The ACL remnant tissue harvested within 90 days after injury predicted higher cell fractions of CD34+, CD44+, CD45+, and CD146+ (Table 2); higher expansion potential at passage 3 and passage 10 (Table 3); and higher potential of osteogenic differentiation in terms of *Osteocalcin* gene expression, ALP levels, and Runx2 (Table 4).

However, there were no significant differences in the osteogenic differentiation-ALP activity (*p* = 0.263), or endothelial differentiation (Tables 4 and 5).

### Age

In the characterization of ACL-derived cells, younger age predicted higher CD34+ levels (Table 2) and higher expansion potential at passage 10 (Table 3).

However, no such correlation was found in stem-like cell fraction, such as CD44+, CD45+, CD146+, CD29+, and Stro-1+ expression (Table 2). In addition, there were no significant differences in osteogenic differentiation or endothelial differentiation (Tables 4 and 5).

### Sex

Female sex was associated with higher ALP activity (Table 4), that attributed to osteogenic differentiation. However, regarding the characterization of ACL-derived cells, there were no significant differences in cell expansion potential or endothelia differentiation potential between male and female participant samples (Tables 2 and 5).

### Combined meniscal and cartilage injuries

No significant differences were detected in the characterization of ACL-derived cells, ALP activity, cell expansion potential or endothelia differentiation potential in the patients who had combined meniscal or chondral injuries (Tables 2-5).

## Discussion

The findings of this study supported the primary hypothesis that the ACL remnant tissues collected within 90 days after injury had higher fractions of CD34+, CD44+, CD45+, and CD146+ cells in the tissue, higher cell expansion potential, and higher osteogenic differentiation potential. These results suggested that a higher healing potential in the ACL remnant tissue could be predicted in the short interval between injury to surgery, and in younger patients.

First of all, surgical timing of ACL reconstruction has widely been discussed in the literatures in terms of risk of meniscal or cartilage lesion [22–26], postoperative range of motion recovery [27–29], incidence of arthrofibrosis [30], and return of quadriceps muscle strength [31]. Besides mechanical stability, the success of ACL reconstruction may also depend on biological graft healing. One study suggested that the ideal time from ACL injury to operation is between 1 and 6 months [32, 33] because of the reduced risk of meniscal or cartilage lesion occurrence [13, 14], better stability, or clinical evaluation [28], and the ability to return to sports and occupations [34, 35]. Meanwhile, Lee et al found there were significantly less mesenchymal stromal cells in the ACL remnant tissue harvested after 6 months of injury [36]. Those results were consistent to our study findings, proving that surgical timing is a critical factor to be considered during medical consultation. Early surgical intervention may preserve the healing potentiality of the ACL remnant tissue, thus predicting a better clinical outcome.

Second, age is another predictor of ACL remnant healing potential. One study found that CD34+ cells were more prevalent in adolescents’ ACL remnants; the number of these cells decreased with age [37]. Our study further proved that younger age was associated with a higher percentage of CD34+ cells in the ACL remnant tissue and higher cell expansion potential. In a recently published cohort study, Nakano et al. [38] proved that ACL-derived cells of younger patients enhanced early bone-tendon healing in an immune-deficient rat model of ACL reconstruction in a rat model.

However, other studies argued that the remnant-preserved augmentation ACL reconstruction did not significantly improve knee stability or clinical scores [39–41]. The conflicts between clinical outcome and basic study results indicated that long-term follow-up, technique improvement, or advanced postoperative function measurements are required to fairly evaluate the remnant preservation ACL reconstruction.

It has been frequently noticed that the delayed ACL reconstruction increased the risks of secondary meniscal and chondral injury [42], and it has been recently reported that delayed ACL reconstruction increased the risks of secondary meniscal and chondral injuries in this population of pediatric patients [33]. Our study also looked into the correlation between the meniscal and articular cartilage injuries and ACL healing potential, but failed to detect the significant difference. Since the risk of meniscal and cartilage injuries was increased with the time after ACL injury, meniscal and cartilage injuries might not be an independent factor to evaluate as a predictor. Further, knee arthrofibrosis is one of the most serious complications that can result from ligament surgery with a reported incidence following ACL reconstruction ranging from 4 to 35 %, and patients with reconstruction within the first two weeks of injury have a significant increase in arthrofibrosis, compared to those surgically treated after three weeks from injury [30]. The early surgical timing may also benefit the patients of less incidence of arthrofibrosis.

This study has several limitations. Some other factors, not detected in this study, may significantly be related to ACL remnant tissue features. The decision of when to undergo ACL reconstruction is likely multifactorial and other factors such as pre-operative status of the knee, family support, school education, work obligation, and mental preparation should also be considered. Further, smoking habits and activity levels were identified as adverse factors affecting ACL reconstruction outcomes. Those factors may modify the intra-articular microenvironment and interact with ACL-derived cells. Another limitation was that a higher percentage of CD34+ does not always correlate with higher differentiation potential of osteogenesis or endothelial, suggesting that CD34+ cells do not switch to mesenchymal cell population with passage, and this problem needs to be addressed in future studies by increasing the sample size.

## Conclusion

In this study, we proved that a shorter interval from injury to surgery resulted in not only a higher fraction of CD34+, CD44+, CD45+, and CD146+ cells in the remnant tissue, but also increased expansion potential and osteogenic differentiation. In addition, we proved that younger age predicted higher CD34+ fractions and higher cell expansion potential. In conclusion, in cases of remnant-preserved ACL reconstruction or ACL reconstruction involving ruptured tissue, early surgical intervention and younger age may predict better healing potential.

## Abbreviations

ACL: anterior cruciate ligament
acLDL: 1,1′-dioctadecyl-3,3,3′,3′-tetramethylindocarbocyanine (DiI)-labeled acetylated low-density lipoprotein
ALP: alkaline phosphatase
APC: allophycocyanin
CD31: cluster of differentiation 31
cDNA: complementary deoxyribonucleic acid
CIs: confidence intervals
DAPI: 4′,6-diamidino-2-phenylindole
DMEM: Dulbecco’s Modified Eagle’s medium
EBM-2: endothelial basal medium-2
EGF: epidermal growth factor
EGM-2: endothelial growth medium-2
FACS: Facial Action Coding System
FBS: fetal bovine serum
GAPDH: Glyceraldehyde 3-phosphate dehydrogenase
OA: osteoarthritis
OR: odds ratios
PBS: phosphate-buffered saline
PDs: population doublings
qRT-PCR: quantitative reverse transcription-real-time polymerase chain reaction
R3-IGF-1: R3 insulin-like growth factor-1
Runx2: Runt-related transcription factor 2
VE-cad: VE-cadherin

## References

[1] Johnson DL, Coen MJ (1995). Revision ACL surgery. Etiology, indications, techniques, and results. Am J Knee Surg.

[2] Middleton KK, Hamilton T, Irrgang JJ, Karlsson J, Harner CD, Fu FH (2014). Anatomic anterior cruciate ligament (ACL) reconstruction: a global perspective. Part 1. Knee Surg Sports Traumatol Arthrosc.

[3] Duthon J, Ménétrey VB, Laumonier L, Fritschy D (2008). “Biological failure” of the anterior cruciate ligament graft. Knee Surg Sports Traumatol Arthrosc.

[4] Harner CD, Baek GH, Vogrin TM, Carlin GJ, Kashiwaguchi S, Woo SL (1999). Quantitative analysis of human cruciate ligament insertions. Arthroscopy.

[5] Crisan M, Yap S, Casteilla L, Chen CW, Corselli M, Park TS (2008). A perivascular origin for mesenchymal stem cells in multiple human organs. Cell stem Cell.

[6] Howson KM, Aplin AC, Gelati M, Alessandri G, Parati EA, Nicosia RF (2005). The postnatal rat aorta contains pericyte progenitor cells that form spheroidal colonies in suspension culture. Am J Physiol Cell Physiol.

[7] Tavian M, Zheng B, Oberlin E, Crisan M, Sun B, Huard J (2005). The vascular wall as a source of stem cells. Ann N Y Acad Sci.

[8] Zengin E, Chalajour F, Gehling UM, Ito WD, Treede H (2006). Vascular wall resident progenitor cells: a source for postnatal vasculogenesis. Development.

[9] Matsumoto T, Kawamoto A, Kuroda R, Ishikawa M, Mifune Y, Iwasaki H (2006). Therapeutic potential of vasculogenesis and osteogenesis promoted by peripheral blood CD 34-positive cells for functional bone healing. Am J Pathol.

[10] Matsumoto T, Ingham SM, Mifune Y, Osawa A, Logar A, Usas A (2012). Isolation and characterization of human anterior cruciate ligament-derived vascular stem cells. Stem Cells Dev.

[11] Mifune Y, Matsumoto T, Kawamoto A, Kuroda R, Shoji T, Iwasaki H (2008). Local delivery of granulocyte colony stimulating factor-mobilized CD-positive progenitor cells using bioscaffold for modality of unhealing bone fracture. Stem Cells.

[12] Tei L, Matsumoto T, Mifune Y, Ishida K, Sasaki K, Shoji T (2008). Administrations of peripheral blood CD34-positive cells contribute to medial collateral ligament healing via vasculogenesis. Stem Cells.

[13] Mifune Y, Matsumoto T, Ota S, Nishimori M, Usas A, Kopf T (2012). Therapeutic potential of anterior cruciate ligament-derived stem cells for anterior cruciate ligament reconstruction. Cell Transplant.

[14] Matsumoto T, Kubo S, Sasaki K, Kawakami Y, Oka S, Sasaki H (2012). Acceleration of tendon-bone healing of anterior cruciate ligament graft using autologous ruptured tissue. Am J Sports Med.

[15] Matsumoto T, Kuroda R, Matsushita T, Araki D, Hoshino Y, Nagamune K (2014). Reduction of tunnel enlargement with use of autologous ruptured tissue in anterior cruciate ligament reconstruction: a pilot clinical trial. Arthroscopy.

[16] Hu J, Qu J, Xu D, Zhang T, Zhou J, Lu H (2014). Clinical outcomes of remnant preserving augmentation in anterior cruciate ligament reconstruction: a systematic review. Knee Surg Sports Traumatol Arthrosc.

[17] Kazusa H, Nakamae A, Ochi M (2013). Augmentation technique for anterior cruciate ligament injury. Clin sports Med.

[18] Muneta T, Koga H, Ju YJ, Horie M, Nakamura T, Sekiya I (2013). Remnant volume of anterior cruciate ligament correlates preoperative patients’ status and postoperative outcome. Knee Surg Sports Traumatol Arthrosc.

[19] Zheng B, Cao B, Li G, Huard J (2006). Mouse adipose-derived stem cells undergo multilineage differentiation in vitro but primarily osteogenic and chondrogenic differentiation in vivo. Tissue Eng.

[20] Zuk PA, Zhu M, Mizuno H, Huang J, Futrell JW, Katz AJ (2001). Multilineage cells from human adipose tissue: implications for cell-based therapies. Tissue Eng.

[21] de Faria AP, Fontana V, Modolo R, Barbaro NR, Sabbatini AR, Pansani IF (2014). Plasma 8-isoprostane levels are associated with endothelial dysfunction in resistant hypertension. Clin Chim Acta.

[22] Wilson WJ, Scranton PE (1990). Combined reconstruction of the anterior cruciate ligament in competitive athletes. J Bone Joint Surg Am.

[23] Wasilewski SA, Covall DJ, Cohen S (1993). Effect of surgical timing on recovery and associated injuries after anterior cruciate ligament reconstruction. Am J Sports Med.

[24] Jee WH, McCauley TR, Kim JM (2004). Magnetic resonance diagnosis of meniscal tears in patients with acute anterior cruciate ligament tears. J comput Assist Tomogr.

[25] Vlychou M, Hantes M, Michalitsis S, Tsezou A, Fezoulidis IV, Malizos K (2011). Chronic anterior cruciate ligament tears and associated meniscal and traumatic cartilage lesions: evaluation with morphological sequences at 3.0 T. Skeletal Radiol..

[26] Yoon JP, Chang CB, Yoo JH, Kim SJ, Choi JY, Choi JA (2010). Correlation of magnetic resonance imaging findings with the chronicity of an anterior cruciate ligament tear. J Bone Joint Surg Am.

[27] Bottoni CR, Liddell TR, Trainor TJ, Freccero DM, Lindell KK (2008). Postoperative range of motion following anterior cruciate ligament reconstruction using autograft hamstrings: a prospective, randomized clinical trial of early versus delayed reconstructions. Am J Sports Med.

[28] Marcacci M, Zaffagnini S, Iacono F, Neri MP, Petitto A (1995). Early versus late reconstruction for anterior cruciate ligament rupture. Results after five years of followup. Am J Sports Med.

[29] Petersen W, Laprell H (1999). Combined injuries of the medial collateral ligament and the anterior cruciate ligament. Early ACL reconstruction versus late ACL reconstruction. Arch Orthop Trauma Surg.

[30] DeHaven KE, Cosgarea AJ, Sebastianelli WJ (2003). Arthrofibrosis of the knee following ligament surgery. Instr Course Lect..

[31] Shelbourne KD, Foulk DA (1995). Timing of surgery in acute anterior cruciate ligament tears on the return of quadriceps muscle strength after reconstruction using an autogenous patellar tendon graft. Am J Sports Med.

[32] Francis A, Thomas RD, McGregor A (2001). Anterior cruciate ligament rupture: reconstruction surgery and rehabilitation. A nation-wide survey of current practice. Knee.

[33] Anderson AF, Anderson CN (2015). Correlation of meniscal and articular cartilage injuries in children and adolescents with timing of anterior cruciate ligament reconstruction. Am J Sports Med.

[34] Karlsson J, Kartus J, Magnusson L, Larsson J, Brandsson S, Eriksson BI (1999). Subacute versus delayed reconstruction of the anterior cruciate ligament in the competitive athlete. Knee Surg Sports Traumatol Arthrosc.

[35] Finsterbush A, Frankl U, Matan Y, Mann G (1990). Secondary damage to the knee after isolated injury of the anterior cruciate ligament. Am J Sports Med.

[36] Lee DH, Ng J, Chung JW, Sonn CH, Lee KM, Hai SH (2014). Impact of chronicity of injury on the proportion of mesenchymal stromal cells derived from anterior cruciate ligaments. Cytotherapy.

[37] Uefuji A, Matsumoto T, Matsushita T, Ueha T, Zhang S, Kurosaka M (2014). Age-related differences in anterior cruciate ligament vascular-derived cells. Am J Sports Med.

[38] Nakano N, Matsumoto T, Takayama K, Matsushita T, Araki D, Uefuji A (2015). Age-dependent healing potential of anterior cruciate ligament remnant-derived cells. Am J Sports Med..

[39] Hong L, Li X, Zhang H, Liu X, Zhang J, Shen JW (2012). Anterior cruciate ligament reconstruction with remnant preservation: a prospective, randomized controlled study. Am J Sports Med.

[40] Yoon KH, Bae DK, Cho SM, Park SY, Lee JH (2009). Standard anterior cruciate ligament reconstruction versus isolated single-bundle augmentation with hamstring autograft. Arthroscopy.

[41] Maestro A, Suárez-Suárez MA, Rodríguez-López L, Villa-Vigil A (2013). Stability evaluation after isolated reconstruction of anteromedial or posterolateral bundle in symptomatic partial tears of anterior cruciate ligament. Eur J Orthop Surg Traumatol.

[42] Csintalin RP, Inacio MC, Funahashi TT, Maletis GB (2014). Risk factors of subsequent operations after primary anterior cruciate ligament reconstruction. Am J Sports Med.

